# Actinic keratosis underlying cutaneous horn at an unusual site—a case report

**DOI:** 10.3332/ecancer.2013.376

**Published:** 2013-11-26

**Authors:** Pragya A Nair, Arvind H Chaudhary, Malay J Mehta

**Affiliations:** Department of Dermatology, Pramukhswami Medical College, Karamsad, Gujarat 388 325, India

**Keywords:** cutaneous horn, cornucutaneum, actinic keratosis

## Abstract

Cutaneous horns are usually found on chronic sun-damaged skin. A cutaneous horn is a rare tumour, often conical, circumscribed, and composed of dead keratin usually derived from base keratinocytes. It occurs mainly in association with underlying benign, premalignant, and malignant cutaneous diseases. The commonest malignancy is squamous cell carcinoma. Thus, to accurately ascertain the nature of the condition at the base of the lesion and to rule out malignancy, an excision biopsy is indicated. Here, we report a case of cutaneous horn over the palmar aspect of the left middle finger in a 45-year-old man whose histopathology showed actinic keratosis.

## Introduction

A cutaneous horn is a conical projection of hard hyperkeratotic excrescence of cohesive keratin projecting over skin. Although it grossly resembles an animal horn, it lacks a bony core. Cutaneous horns are found in the upper parts of the body, such as the face, neck, and shoulders, and are frequently related to actinic damage [1]. Cutaneous horns are thought to result from underlying benign, premalignant, or malignant in 61.1%, 23.2%, and 15.7% of cases, respectively [2]. Cutaneous horns have been noted on top of many clinical conditions such as keratoacanthoma, actinic keratosis, warts, molluscum contagiosum, seborrheic keratosis, Bowen’s disease, malignant melanoma, basal cell carcinoma, or squamous cell carcinoma [3].

We hereby report a case of a cutaneous horn at an unusual site, that is, the palmar aspect of the middle finger, with underlying histopathologic changes of actinic keratosis.

## Case report

**Figure 1:**
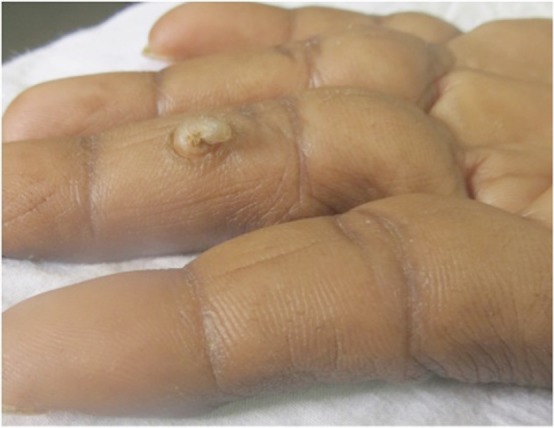
A horny growth of around 2 cm in length with a broad base over the palmar aspect of the middle phalanx of the left middle finger.

A 45-year-old man presented with a single lesion over the palmar aspect of the middle phalanx of the left middle finger since 12 months prior. There was no history of trauma or surgery at that site in the past. No itching, pain, or bleeding was present. The patient was a farmer by occupation. No significant family history was present. On examination, a firm, horny, and curved growth of around 2 cm in length with a broad base was present over the palmar aspect of middle phalanx of the left middle finger with no erythema at the base (Figure 1). No lymphadenopathy was present. Routine blood investigations, urine examination, and chest X-ray were normal. An excision biopsy was done.

**Figure 2:**
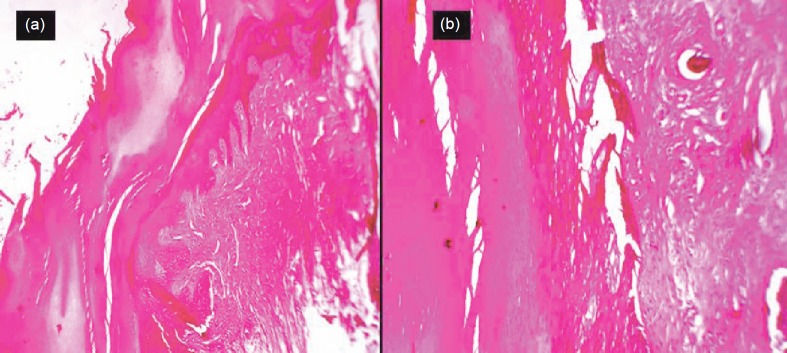
(a) Hyperkeratosis and focal parakeratosis, Hematoxylin & Eosin (H & E) stain 4x; (b) loss of polarity and disordered arrangement of keratinocyte in stratum malphigian with part of normal dermis, H & E stain 10x.

Histopathology showed extensive hyperkeratosis and focal parakeratosis. The epidermis was thickened and showed irregular downward proliferation in dermis (Figure 2a). A varying proportion of keratinocyte in the stratum malphigian showed a loss of polarity and disordered arrangement with part of the normal dermis showing collagen bundles horizontally placed (Figure 2b). A diagnosis of actinic keratosis was given.

## Discussion

Cutaneous horns, or cornucutaneum, are benign, elongated, keratinous projections from the skin, ranging in size from a few millimetres to many centimetres and resembling a miniature horn. The base of the horn may be flat, nodular, or crateriform. All animal horns except those of rhinoceroses contain bone casts that are not seen in cutaneous horns in human beings, which are simply composed of compact keratin. Cutaneous horns may be considered a common entity in the Caucasian population; a study reports 643 patients over a ten-year period with 32 new patients annually [2]. It is usually seen over the face, pina, nose, forearm, and dorsal aspect of the hand [3]. Cases over the areas not exposed to sunlight, such as the penis, mucosal part of the lower lip, and nasal vestibule, have also been reported [4, 5]. Clinically, it is a hard, yellowish-brown horn, often curved, having circumferential ridges surrounded by normal epidermis or acanthotic collarette. Weeson’s defined criteria (1987) for a horn are that it should be straight or curved and 2–2.5 cm long [6].

Classical cutaneous horns have dysplastic epidermal changes similar to solar keratosis with no atypicality or loss of polarity.

The pathogenesis of this abnormal formation of keratinised material has not been fully elucidated. It may be of clinical importance because the underlying condition may be a malignant lesion. It is difficult to define the underlying lesion, especially in superficial biopsies; therefore, deep biopsies or the total excision of small lesions is recommended [7]. Malignancy is present in 16–20% of cases, with squamous cell carcinoma being the most common type [8] in 94% of horns with a malignant base. Among the predisposing factors are advanced age, male sex, and sun exposure [7]. Tenderness at the base of the lesion, large size, wide base, or low height-to-base ratio horns are more likely to display a malignant base [9]. Multiple horns may occur in some patients [10]. Actinic keratoses have been reported as the most common horn base entity (37.4%) in a study of 230 horns [11], but they may also result from seborrheic keratoses, warts, keratoacanthoma, squamous cell carcinoma, basal cell carcinoma. Extremely rare cases associated with metastatic renal cell carcinoma [12], lymphoma [13], dermatofibroma [14], and pyogenic granuloma [15] have been reported. The base of the horn will display the characteristic feature of the pathologic process responsible for the development of the horn [16].

Actinic keratosis is a premalignant condition. The lesion starts as a flat scaly lesion and later grows into a large wart-like area. It commonly ranges from between 2 and 6 mm in size, may be dark, light, tan, pink, red, or a combination of all these shades. It occurs on sun-exposed areas of the body such as face, ears, neck, scalp, chest, back of hands, forearms, and lips. Up to 20% of untreated actinic keratosis can progress to malignancy. Histologically there is thickened stratum corneum with scattered areas of parakeratosis, associated loss of the granular layer and thickening of the epidermis. The normal ordered maturation of the keratinocytes is disordered to varying degrees, there may be widening of the intracellular spaces, cytologic atypia, such as abnormally large nuclei. The underlying dermis often shows severe actinic elastosis and a mild chronic inflammatory infiltrate. Cutaneous horn that represents actinic keratosis demonstrates similar but more exaggerated changes with massive tiers of hyperkeratosis and parakeratosis.

The unique feature of this case is that the cutaneous horn was located on the palmar aspect of the middle finger, which is an uncommon site. Only one such case has been reported at an unusual site over the right index finger tip, but it also did not show any underlying premalignant or malignant changes [9]. It suggests that trauma might be a predisposing factor for the development of cutaneous horn in areas that are not exposed to actinic damage.

Excision biopsy of the lesion and histopathological examination to rule out malignancy is mandatory. As malignancies should be excised with appropriate margins and evaluated for metastasis, a careful physical examination of the lymph nodes draining the area of lesion is required [17]. Treatment options include wide surgical excision, electrocautry, cryotherapy, carbon dioxide, or NdYAG laser [18].

## Conflicts of interest

The authors have no conflicts of interest to declare.

## References

[1] Bondeson J (2001). Everard Home, John Hunter Cutaneous horn: a historical review. Am J Dermatopathol.

[2] Yu RC, Pryce DW, Macfarlane AW, Stewart TW (1991). A histopathological study of 643 cutaneous horns. Br J Dermatol.

[3] Copcu E, Sivrioglu N, Culhaci N (2004). Cutaneous horns: are these lesions as innocent as they seem to be?. World J Surg Oncol.

[4] Rekha A, Ravi A (2004). Cornucutaneum-cutaneous horn on the penis. Indian J Surg.

[5] Mehmet Mutaf (2007). A rare perioral lesion: cutaneous horn of the lower lip. Eur J Plast Surg.

[6] Gupta A, Jain AK (1997). A cutaneous horn over seborrhoeic keratosis of pinna. Ind J Otolaryngol Head Neck Surg.

[7] Schwartz RA, Bridges TM, Butani AK, Ehrlich A (2008). Actinic keratosis: an occupational and environmental disorder. J Eur Acad Dermatol Venereol.

[8] Solivan GA, Smith KJ, James WD (1990). Cutaneous horn of the penis: its association with squamous cell carcinoma and HPV-16 infections. J Am Acad Dermatol.

[9] Tauro LF, Martis JS, John SK, Kumar KP (2006). Cornucutaneum at an unusual site. Ind J Plast Surg.

[10] Pyne J, Sapkota D, Wong JC (2013). Cutaneous horns: clues to invasive squamous cell carcinoma being present in the horn base. Dermatol Pract Conc.

[11] Schosser RH, Hodge SJ, Gaba CR, Owen LG (1979). Cutaneous horns: a histopathologic study. South Med J.

[12] Ozturk S, Cil Y, Sengezer M, Yigit T, Eski M, Ozcan A (2006). Squamous cell carcinoma arising in the giant cutaneous horns accompanied with renal cell carcinoma. Eur J Plast Surg.

[13] Dasgupta S, Mitra D, Bhattacharya A, Sur P (2006). B cell lymphoma with unusual clinical cutaneaous presentation. J Cancer Res Ther.

[14] Kim YJ, Jeon J, Son SW, Kim AR, Oh CH, Song HJ (2006). Dermatofibroma: unusual lesion with underlying cutaneous horn. Kor J Dermatol.

[15] Findlay RF, Lapins NA (1983). Pyogenic granuloma simulating a cutaneous horn. Cutis.

[16] Mencia-Gutierrez E, Gutierrez-Diaz E, Redondo-Marcos I, Ricky JR, Garcia-Torre JP (2004). Cutaneous horns of the eyelid: a clinicopathological study of 48 cases. J Cutan Pathol.

[17] Kumaresan M, Kumar P, Pai MV (2008). Giant cutaneous horn. Ind J Dermatol.

[18] Lowe FC, McCullough AR (1985). Cutaneous horn of the penis: An approach to management: case report and review of literature. J Am Acad Dermatol.

